# Centenary of Soil and Air Borne Wheat Karnal Bunt Disease Research: A Review

**DOI:** 10.3390/biology10111152

**Published:** 2021-11-09

**Authors:** Mir Asif Iquebal, Pallavi Mishra, Ranjeet Maurya, Sarika Jaiswal, Anil Rai, Dinesh Kumar

**Affiliations:** Centre for Agricultural Bioinformatics, ICAR-Indian Agricultural Statistics Research Institute, New Delhi 110012, India; ma.iquebal@icar.gov.in (M.A.I.); mishrapallavi58@gmail.com (P.M.); ranjeet.maurya04@gmail.com (R.M.); sarika@icar.gov.in (S.J.); anil.rai@icar.gov.in (A.R.)

**Keywords:** genome, pathogen, QTL, quarantine, *Tilletia indica* (Ti), wheat

## Abstract

**Simple Summary:**

The present review provides a comprehensive, in-depth, and updated information on Karnal bunt by summarizing scientific discoveries of this disease from different sources/literature of the past 100 years or more. Consequently, herein we looked at the current situation of the bunt pathogen of wheat. The review reveals the importance of next generation sequencing (NGS)-based genomic studies in Karnal bunt, from assembly to genome-wide association studies (GWAS), its diagnostics and management. The compiled exhaustive information can be beneficial to the wheat breeders for better understanding of the incidence of disease and near-future solutions to control the Karnal bunt disease of wheat.

**Abstract:**

Karnal bunt (KB) of wheat (*Triticum aestivum* L.), known as partial bunt has its origin in Karnal, India and is caused by *Tilletia indica* (Ti). Its incidence had grown drastically since late 1960s from northwestern India to northern India in early 1970s. It is a seed, air and soil borne pathogen mainly affecting common wheat, durum wheat, triticale and other related species. The seeds become inedible, inviable and infertile with the precedence of trimethylamine secreted by teliospores in the infected seeds. Initially the causal pathogen was named *Tilletia indica* but was later renamed *Neovossia indica*. The black powdered smelly spores remain viable for years in soil, wheat straw and farmyard manure as primary sources of inoculum. The losses reported were as high as 40% in India and also the cumulative reduction of national farm income in USA was USD 5.3 billion due to KB. The present review utilizes information from literature of the past 100 years, since 1909, to provide a comprehensive and updated understanding of KB, its causal pathogen, biology, epidemiology, pathogenesis, etc. Next generation sequencing (NGS) is gaining popularity in revolutionizing KB genomics for understanding and improving agronomic traits like yield, disease tolerance and disease resistance. Genetic resistance is the best way to manage KB, which may be achieved through detection of genes/quantitative trait loci (QTLs). The genome-wide association studies can be applied to reveal the association mapping panel for understanding and obtaining the KB resistance locus on the wheat genome, which can be crossed with elite wheat cultivars globally for a diverse wheat breeding program. The review discusses the current NGS-based genomic studies, assembly, annotations, resistant QTLs, GWAS, technology landscape of diagnostics and management of KB. The compiled exhaustive information can be beneficial to the wheat breeders for better understanding of incidence of disease in endeavor of quality production of the crop.

## 1. Introduction

The Karnal bunt (KB), also named “partial bunt” or “new bunt” caused by *Tilletia indica* (syn. *Neovossia indica* (Mitra) Mundkur) is a soil, seed or air-borne wheat disease which was described first as a bunt of wheat in the year 1909 [1], but was first observed by Mitra in April 1930. The disease is caused by the heterothallic fungus *Tilletia indica* (Ti) which is a pathogen that reproduces sexually after teliospores have germinated. Mitra reported this bunt on wheat (*Triticum aestivum* L.) in 1931 in experimental seeds grown at the Botanical Station, Karnal, Haryana in India. From here only, the name Karnal bunt originated. The seeds become inedible/unpalatable, inviable and infertile with precedence of trimethylamine secreted by teliospores in infected seeds. Mitra was of the view that the pathogen was present much earlier and might have been the one identified by Howard and Howard in 1909. Its incidence had grown drastically since late 1960s from north western India to northern India in the early 1970s. Initially, in 1931, Mitra identified the causal pathogen of KB as *Tilletia indica*, thereafter known by the preferred scientific name *Tilletia indica* Mitra [2], but later Mundkur renamed it *Neovossia indica* (Mitra) Mundk in 1940 [3]. The infested grains/ spikelet becomes black with powdered smelly spores which can be dispersed to distant places. Dangerously, the spores remain viable for years in soil, wheat straw and farmyard manure as primary sources of inoculum. These spores are conducive for growth at 15–25 °C temperature and humid soil. The distribution of this pathogen is very restricted, mostly to the Indian subcontinent and to a comparatively lesser extent to Mexico and the southwestern USA. In the regions where the cultivars that were highly susceptible to Karnal Bunt were grown in India, losses of up to 40% were reported. However, the overall national loss due to Karnal Bunt was nearly 0.5%. Mexico reported yield loss due to KB at the maximum 1%. The cumulative reduction of national farm income in USA due to KB was USD 5.3 billion (2003–2007) [4]. Over the century, it was initially referred to as new bunt [2] and later as Karnal bunt [3], or partial bunt [5] of wheat in various reports.

The pathogen *Tilletia indica* (Ti) mainly affects common wheat, durum wheat, triticale and other related species. The quality but not the quantity is affected by this pathogen. It is less vulnerable to the triticale. The pathogen of Karnal bunt is seed, soil and air borne. In nature, the fungus is heterothallic and develops haploid secondary sporidia. Compatible spores fuse the opposite mating types together to produce dikaryotic hyphae that infect the wheat heads. Through the germinal end, the fungus reaches the wheat grains and partially turns the seeds into sori filled with stinking teliospores. This affects the seed quality and crop yield.

Wheat, being the main cereal, is grown worldwide. In India, it has a significant contribution in the country’s agricultural economy. The crop is mainly grown in the northern belt of Uttar Pradesh, Punjab and Haryana states, contributing 55% of total area and 67% of total production [6,7]. In wheat growing areas, the major concern is the diseases that lead to reduction in productivity. In India, wheat is affected by various diseases, like rusts, powdery mildew, loose smut, leaf blight and Karnal bunt [8], among which, Karnal bunt is the prime disease. The prevalence of Karnal bunt disease in the main wheat-growing countries lead to reduced wheat production due to poor grain quality and the fishy odor which is due to the presence of teliospore-secreted trimethylamine. It had been a minor disease previously found only in northwest India but was unusually widespread in Northwest India during the 1969–1970 crop seasons. Since 1974–1975, Karnal bunt has spread from West Bengal to the western border, all over Northern India. Reportedly, the disease occurs in many parts of the world such as Pakistan, Nepal, Iran, Iraq, Afghanistan and Mexico. Several varieties of Indian wheat have been reported to be resistant to Karnal bunt but most of the cultivars are still susceptible [9,10]. KB is a major limiting factor in the export of wheat, as most countries control this pathogen as a quarantine pest. It threatens wheat trade between countries as a seed borne pathogen and has relevance for seed certification [11,12]. It decreases the quality of seeds, alters the chemical composition of the infected grains and makes the seed inedible. Wheat with 3% of bunted grain is not appropriate for human consumption [13]. Highly infected grains show substantial decrease in viability of the crop. In recent years, the incidence of the KB disease in the wheat industry has become a major concern. The current impact of KB needs to be updated since its inception which is lacking. We analyzed more than a hundred reviews/literature and research studies related to KB to present the overview of its research published during past 100 years, since 1909. We also explored and compiled the genomic level advancement applied for developing the KB management strategies.

## 2. Pathogen Biology

In 1944, Mundkur renamed the genus of KB pathogen from “*Tilletia*” to “*Neovossia*”, based on the large number of nonfusing primary sporidia made. However, Fischer again started referring the pathogen as *Tilletia Indica* [14]. The genus *Tilletia*, belongs to the family Tilletiaceae, order Ustilaginales and class Basidiomycotina. There are approximately 140 known species of genus *Tilletia*, all of which cause diseases on grass hosts belonging to the family Poaceae. *Tilletia indica* Mitra (syn. *Neovossia indica* (Mitra) Mundkur) belongs to the Class of Basidiomycetes, Order Ustilaginales and Family Tilletiaceae. The pathogen is heterothallic and reproduces sexually after teliospores have germinated. Primary and secondary sporidia or hyphae as compatible mating types must fuse to produce dikaryon that increases the chances of variation due to heterozygosity, which plays an important role in developing new variants [15]. The fungus infects the ovaries in the developing heads of wheat and transforms the seed to a dark colored powdery mass consisting of millions of teliospores [16,17]. The most effective carrier of pathogens for inter-regional and long-range spore dispersal is infected seeds [18].

## 3. Lifecycle

Mitra [1] reported the pathogen to be soil borne, but now it is recognized as a seed borne and air borne disease as well. It is not transmitted directly from seed to the plant. The pathogen is airborne in the form of teliospores or sporidia. The lifecycle starts when teliospores germinate and develop primary sporidia, present on the soil surface. The extent of disease that occurs in or on the host depends on the pathogen’s presence in sufficiency, a susceptible host and a favorable environment. The fungus is heterothallic in nature and during its lifecycle, it undergoes a transition from monokaryotic to dikaryotic developmental stages. There is a wide genetic variation due to heterothallism among pathogens, which is due to the fusion in opposite mating types of sporidia and thus influence on virulence behavior. Mating between two compatible Ti allantoids sporidia and the resulting genetic recombination induces substantial genetic and pathogenic variability during the onset of disease. The primary sporidia lead to secondary sporidia through bud formation, which continually multiply on the surface of the host leaf and pass from lower to upper leaf surfaces by monkey jumping. The secondary sporidia are air-borne to plant surfaces where they can germinate to produce additional generations. They infect the stage between the boot leaf and the soft mass.

**Figure 1:**
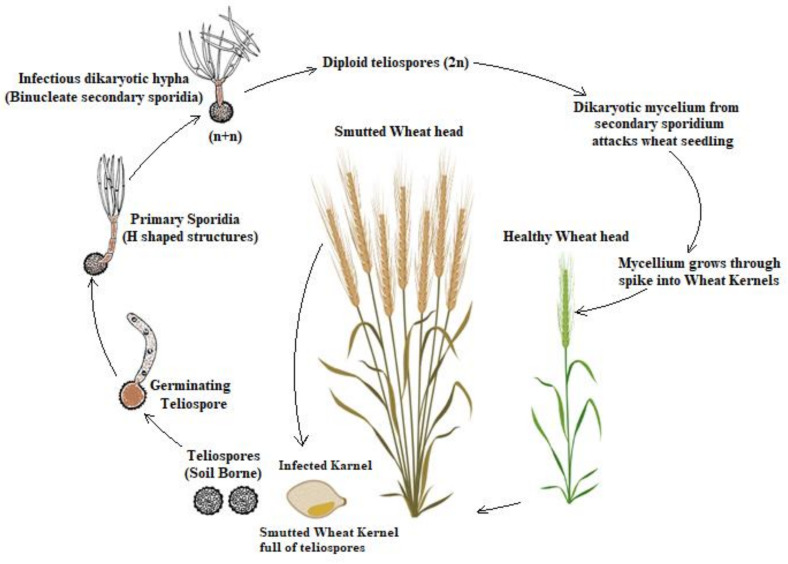
Disease cycle of the causal pathogen, *Tilletia indica* (Ti).

The mature teliospore is in a diploid (2n) form, and meiotically after germination, divides the nucleus and then it produces the haploid primary sporidia mitotically. Fuentes and Duran [19] reported that meiosis happens during germination, followed by a series of mitosis in teliospores and promycelia, forming multiple haploid nuclei in the promycelia. The disease cycle of this pathogen occurs in three morphologically distinct forms, as shown in Figure 1. These forms are: a haploid nonpathogenic phase that grow as sporidal form. Primary sporidia germinates to produce an infectious and reproductive entity, i.e., allantoid and filiform secondary sporidia, respectively; filamentous dikaryon which is produced after the fusion of two pathogenic compatible haploid cells that colonize in the plant tissues; diploid teliospore that resulted from karyogamy in sporogenous mycelium in host tissues. Then, the secondary sporidia arrive on host florets during the germination and penetrate its lemmas, glumes, paleas and rachis via stomata. The originated spores are capable of surviving in soil, wheat straw and manure in farmyards for many years [20].

## 4. Karnal Bunt Disease Distribution over the Century

The pathogen Ti has a restricted distribution, mostly confined to the Indian subcontinent and a small region of Mexico and southwestern America. KB is not widespread outside the northwest region in the Indian subcontinent. This disease has been officially recognized in countries like India, Iran, Afghanistan, Iraq, Pakistan, Mexico, Nepal, United States of America. KB was first described as a bunt of wheat from Lyallpur (Faisalabad), Pakistan in the year 1909 [1] and thereafter first detected in northern India’s Karnal district in 1931. The disease was soon found in several other regions across north and central India, and is now widespread in many other Asian and Middle Eastern countries. In east and central India the incidence of disease is very low. KB has never been recorded in southern or northeastern India [21,22,23]. After Mitra’s first study in 1931, in 1934 McRae documented KB in a virulent form at Karnal.

The disease was later discovered in the Sindh Province of Pakistan in 1941 and the Delhi state of India. In 1943, it was widespread in the North West Frontier and Punjab Provinces of Pakistan [3,24]. The incidence was low during 1944–1945 [25], but in 1948, severe damage by KB was observed in Pakistan’s North West Frontier and Punjab Provinces [5]. Though this disease originated in Southern Asia, it was later reported in Nepal, Iran, Afghanistan, Syria, Iraq, Mexico and America. The disease remained less destructive until the 1970s, but thereafter serious outbreaks began to occur which coincided with the shift to irrigated, semidwarf agriculture with high fertilizer use.

KB was also confirmed in Iraq [26], Southern Nepal [27] and Southern Iran [28]. In Lebanon, Syria, Sweden and Turkey [29], the Ti teliospores were found in wheat germplasm, but signs of the disease were never reported. Hence, in these countries, the status of the disease remained unclear. Later, the subsequent shipments of wheat germplasm from Turkey, Lebanon and Syria were free of contamination with Ti teliospores [30]. Further, the disease was established in the north west Mexico [31] and recorded in Arizona, Texas and Southern California in the United States [32]. Findings by Da Luz et al. [33] reported teliospore contamination in a seed lot produced in 1989 at Rio Grande do Sul State, Brazil. However, no further report has been published since then. KB was also found in a region of the South African Northern Cape Province [34], in addition to on the subcontinent of India including India, Pakistan, Nepal and Afghanistan. The incidence of Karnal Bunt has gradually increased across India since the mid-1970s, especially in the Punjab and Haryana [35].

Karnal Bunt was not detected outside Asia until 1972, when it was reported from Sonora state in Northern Mexico. At that time, the disease was confined to Sonora’s Yaqui and Mayo valleys, and was present only in small quantities in the farmers’ fields. Disease surveys in these valleys, however, showed KB infestation on 64% of farms in the early 1980s. KB’s first study of durum wheat in the US was confirmed in Arizona on 8 March 1996, and after a few weeks, bread wheat was also reported with KB in California. Sample seeds of harvested wheat collected by the Arizona Department of Agriculture were tested for the presence of teliospores in 1993, which suggested that the disease appeared in Arizona in 1992. It was soon discovered that infested seeds were also shipped and planted in Texas and New Mexico. In Texas and New Mexico, fields that were planted with infected seeds had been destroyed and examined for many years. Thereafter, it was reported in a grain sample from San Saba County in central Texas in September 1997 through a National Survey. Disease occurrence was clustered in California, Arizona and Texas and limited to relatively small areas. Subsequent examination by APHIS around Young County, of grain samples resulted in detection of pathogen in the neighboring Archer, Baylor and Throckmorton counties. Countries like Canada, the United States, Russia, China and Australia were also at risk of transmitting the KB disease.

These reports emphasize that the disease has long been prevalent in the subcontinent, infecting native wheat grown in northwestern India, but never caused significant yield losses. The KB remained confined to the Punjab, Jammu and Tarai regions of Uttar Pradesh until 1974–75 [36]. As there were no strict regulatory restrictions on wheat movement in India, the disease spread to more areas in the wheat belt of northwest regions. Karnal bunt has been then reported in the Haryana, Punjab, Jammu, lower Himachal Pradesh, Rajasthan, Delhi, Uttar Pradesh and Bihar states [21]. Thereafter [37] also recorded the presence of KB in West Bengal, Madhya Pradesh, Bihar and Gujarat, which shows KB covering the eastern, central and western India. The regions of Madhya Pradesh, southern Rajasthan, Maharashtra, Odisha, Assam, Meghalaya, Karnataka, Andhra Pradesh, Tamil Nadu and Kerala are free of KB because of the high temperatures in these areas [38].

**Figure 2:**
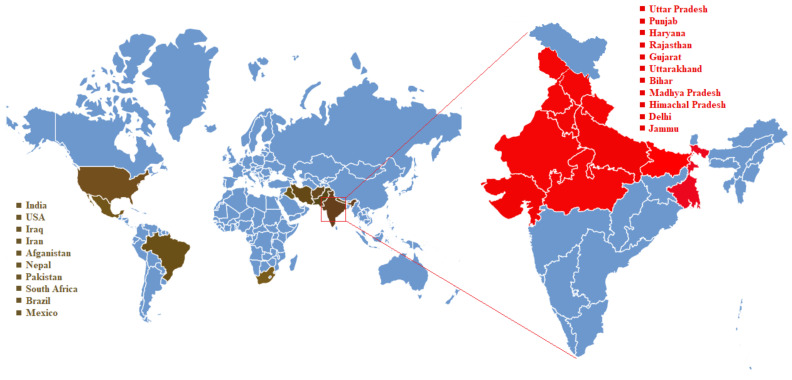
Countries with incidence of Karnal Bunt disease as reported till 2020. Distribution of infected countries (shown in brown) and states within India (shown in red). The KB positive states are indicated in red as per survey report of ICAR-IIWBR, Karnal, 2019).

In the Bihar and West Bengal states of India, the incidence of KB significantly decreased comparatively with high incidence area of Punjab, Jammu region, lower Himachal Pradesh, Haryana, Uttar Pradesh and Delhi. Extensive postharvest surveys were conducted for monitoring KB incidence in the northwest regions of India, where the incidence of Karnal bunt is relatively high and various Indian Wheat-growing states such as Haryana, Punjab, Rajasthan, Himachal Pradesh, Uttarakhand, Uttar Pradesh, M.P., Gujarat, Maharashtra, and Karnataka (IIWBR Annual-Report-2018–2019) [39]. Samples monitored from Gujarat, M.P., Karnataka and Maharashtra were found free of KB infection among various states. The KB infestation in the country as a whole was 21.8%. The Haryana samples showed highest (49.5%) infection followed by Rajasthan (49.0%) and Punjab (28.2%). Jammu, Punjab, Haryana, Uttarakhand, Himachal Pradesh, Rajasthan and Uttar Pradesh states of India also shows the KB incidence during 2017–2018 crop season (Figure 2).

## 5. Impact of the Dynamics of Climate Change on Disease Development

Environmental factors play an important role in the development of disease. For infection and disease development, KB pathogenesis is heavily dependent on favorable weather conditions. Under environmental circumstances, teliospores can live for several years in the extremes of desert and cold climates. Five years of teliospore survival has been recorded under intense environmental stress conditions in the laboratory [20]. The optimal conditions for disease infection that promotes KB development are high relative humidity, cloudiness with mild temperatures and rainfall during anthesis. Dry weather, sunshine and high temperatures had adverse influence on the disease development. A temperature range of 15–25 °C and humidity are ideal for teliospore germination in soil. Additionally, the different predictive regression models have been introduced to forecast KB wheat disease and conclude the relative humidity of 70% favorable for growth and development of teliospore. In addition, daytime temperatures between 18–24 °C and soil temperatures in the range of 17–21 °C increase KB severity and are correlated with KB sporidial showering, rendering favorable the KB infection climate for wheat. In Northern India, this condition is usually prevalent during February to March [32]. Teliospore helps the pathogen to survive during the hot dry summer months of May and June, when the maximum temperature reaches up to 45 °C. During harvesting, the teliospores fall, spreading both in the air and on the soil surface and giving rise to new infections in the next season when the environmental conditions become favorable [38]. Moisture changes with changing climate have an effect on both wheat and pathogen. Increased moisture promotes teliospores germination in the absence of the host. The shift in climate variability and humidity levels to the temperature has a high correlation with the severity of the disease from the emergence of the flag leaf to the milky stage [40]. A favorable association between the host-pathogen–environment is the cause of the occurrence of KB disease. Hence, the changes in environmental conditions in areas where KB disease occurs would also have an effect on its presence and severity. Some of the expected climate changes influencing the severity of the KB pathogen include increased temperature, precipitation changes increased CO_2_ level and drought [41]. It has been suggested that an increase in temperature may increase wheat rust infection. Many models of climate change have projected that teliospore survival and germination could theoretically be favorable and this could lead to increased infection and pathogen growth. A rise in CO_2_ levels increases leaf and stem growth rates, resulting in relatively dense canopies with high humidity promoting KB disease. Rains can extend the pathogen’s habitat to other grass hosts that it cannot naturally infect [31]. Higher CO_2_ can reduce the rate of decomposition of plants, resulting in increased crop residue that can lead to an epidemic of disease. With a shift in CO_2_ concentration, temperature and humidity level, the activity of chemical fungicides would decrease in efficacy due to decreased uptake. The change in temperatures and CO_2_ levels may contribute to accelerating the pathogen’s major evolutionary process by making the environment more optimal.

## 6. The Disease Impact on Economic Extent

Karnal bunt disease of wheat has high impact on quality, and not on yield loss. The losses caused by KB differ greatly from one country to another. Karnal bunt has existed in the country since 1930, but no significant economic consequences are evident from the disease. KB can be a serious disease and there have been reports of casualties ranging from 5–20%. Even in years of extreme epidemics in India, total wheat crop damage ranged from 0.3–0.5% of the total yield. However, surveys conducted in India during the heavy disease incidence years showed a total loss of 0.5%. The annual yield loss recorded by Munjal et al. [42] was of 0.2% in the Jammu & Kashmir, and Punjab states. The overall damage to wheat crops was just 0.2–0.5% of total production during the worst years of the epidemic [21]. In the most extreme years from 1982 to 1989, especially in Uttar Pradesh, Singh [43] reported 0.3–0.5% yield loss. Hassan [44] reported grain losses from Pakistan of 2–3%. The economic losses in Mexican states affected from Karnal bunt of wheat calculated by Fuentes-Davila [45], were 2.0%, 2.1% and 0.3% in Sinola, Sonora and South Baja California, respectively. Direct losses in terms of quality and seed exports were estimated in northwest Mexico at 0.12% per year. The overall losses, including direct and indirect due to disease in Mexico are therefore projected to be USD 7.02 million/year. The quarantine-related procedural disputes create barriers to trade beyond reducing tonnage and losing grain quality. Hence, the losses of yield mentioned in the literature vary from the ~1% to 20%. However, it is disputed by scientists and hence it is not possible to be completely conclusive at this point about possible yield losses.

## 7. Technology Landscape of KB Detection

Wheat KB pathogen identification is mainly based on traditional methods such as direct dry seed examination, soaking tests, washing tests, incubation tests, embryo count tests, blotter tests and filter-centrifuge extraction techniques [46]. Until the end of the 20th century, these techniques were in common practice. For accurate and rapid detection of KB pathogen, molecular approaches were established that are precise, rapid and active [47]. After the introduction of the Polymerase chain reaction (PCR) technique for KB disease, some molecular diagnostic methods were developed. In recent decades, genomic methods for the characterization of the KB pathogen have significantly simplified and enhanced the detection and identification of Ti.

### 7.1. Morphological Identification

There exist a number of methods to distinguish Karnal bunt from other pathogens. Microscopic analysis, isozyme analysis and pathogenicity tests are among the grain testing methods for the presence of KB spores. These procedures have disadvantages because they depend on individual examiners and are therefore totally subjective. Conventional methods prevail that include morphological contrast with other species of *Tilletia* as morphologically identical teliospores found on stored or harvested wheat which can be misidentified as Ti (Table 1). Therefore, DNA-based molecular methods have been useful in the identification of similar teliospores.

### 7.2. Molecular Approaches of Identification

#### 7.2.1. Biophysical Techniques

A good diagnostic method can be spectrophotometeric scans of extracts of Ti in the ultraviolet range. Additionally, photoacoustic spectroscopy is a technique for detecting the output of light-induced heat after the sample absorbs pulsed radiation. The photoacoustic spectrum of six pathogens for the identification and differential diagnosis analysis of dry spores, *viz.* Ti, *Ustilago tritici*, *Tilletia barclayana*, *Helminthosporium sativum*, *Alternaria triticina* and *Ustilaginoidea virens* were created using an indigenous photoacoustic spectrometer in the wavelength range of 200 to 800 nm with the modulation frequency of 18 Hz [48]. The outcome peaks intensity obtained from pathogens in the photoacoustic spectrum were matched, which showed that a few additional peaks were observed for others.

#### 7.2.2. Isozyme Based Techniques

Isozymes are the enzymes having same specificity of the substrate but different kinetics and molecular weights of the enzymes. In order to analyze the genetic variation by taxonomy, an isozyme method was performed [49]. The isozyme electrophoresis profile of starch gel was used by Bonde et al. [50], to distinguish between Ti and *Tilletia Barclayana*. The use of such experimental exercise is tedious, and requires specialized knowledge.

#### 7.2.3. DNA-Based Techniques

For the identification of low amounts of seed-borne pathogens, DNA-based techniques are highly susceptible. We can detect 2–3 cells/mL of initial seed washing by this method. Together with six short random sequence primers, a polymerase chain reaction was used to conduct RAPD profile of seven wheat and rice, seed-borne fungal pathogens that exhibited different polymorphic DNA. Efforts were made to develop an effective molecular method to classify Ti teliospores using Ti 17 M1/M2 (825 bp) and Ti 57 M1/M2 (118 bp) BIO-PCR and primer pairs. There could be as few as 5 teliospores detected from per 50 g of seeds of wheat [51]. In this case, the PCR-based technique is important because KB teliospores have similar morphology to other *Tilletia* species. TaqMan probes for Karnal bunt of wheat have been used to establish many real-time PCR assays [11].

#### 7.2.4. Immunological Techniques

This has been most adapted method used in diagnosis to identify plant pathogens [52]. This approach is the most reliable and is also effectively used for disease detection and differential diagnosis. The advent of enzyme-linked immunosorbent assay and highly selective monoclonal antibodies has made immunoassays as standard technique for detecting antibody binding sites or antigens that are unique to the pathogen, where the relevant antigens are covalently bound independently to 0.5 μm or 0.9 μm diametric plastic microspheres. Antigen linked spheres were subsequently exposed to standard serum or particular antibody-containing serum.

#### 7.2.5. ELISA

ELISA test for fungi contains fungal antigen, especially glycoproteins of the test sample in microtiter wells. The “tag” enzyme was used to produce colored substance for binding of antigen and specific antibody which works on its substrate. Varshney et al. [53], reported microtiter ELISA used for the early identification of Karnal bunt pathogen when infection rates are very low for fungal biomass determination in infected tissues based on their pattern of immune reactivity. Further, this technique was useful to monitor the antigenic properties during the sexual production of KB pathogen along with the recognition of pathogens’ genetic diversity [54].

#### 7.2.6. Tissue Imprinting

In tissue print immunoblot process, a direct blotting of plant tissue has been done into the nitrocellulose membrane [55,56]. A process called seed immunoblot binding assay was developed to measure the teliospore load on seeds in the vigor testing [57]. In this, the teliosporic antigens of the infected seeds are adsorbed and diffused as per the teliosporic load on the nitrocellulose membrane. The antiteliosporic antibody immunoprocessed sheet forms the colored imprint of severity of Karnal bunt infection for the bunted grains.

#### 7.2.7. Immunofluorescence Assays

In this technique, fluorescent dye molecules are used (usually rhodamine isothiocyanate or fluorescence isothiocyanate), which conjugate with pathogen-specific antibodies. In fluorescence microscope, antigens become visible those are bound to microscope slides. This detects the teliospores of infected wheat seeds present in sori. The technique is based on the indirect ELISA concept in which polyclonal antibodies are released against surface telisoporic antigens. Under the microscope, the teliospores are seen as bright green and ring-shaped fluorescence. This technique supports in understanding the molecular basis of teliospore germination [48].

#### 7.2.8. Immunodipsticks and Dot Blot

The immunodipstick/dot blot assay test for KB diagnostics has the same procedure as of ELISA. It uses plastic tags or cards coated with nitrocellulose or polyvinylidene difluoride. Due to the charged nature of the nitrocellulose membrane, teliosporic antigens are adsorbed. Immuno-processing of dipsticks by antiteliospore antibodies results in color production which shows the pathogen presence. It can predict tiny proportion (5 ng of antigen) of teliospores [58].

#### 7.2.9. Agglutination Assay

This technique is best suitable (more cost-effective, faster and easier to use) for the detection of solubilized teliospore antigens, where the sonic and detergent extraction are used to solubilize the teliospore antigen. It allows the antigen to adsorb on the surface of blue-colored latex beads, which leads to polyclonal antiteliospore serum-based immune-processing. The existence of KB infection is expressed by the positive agglutination response [59].

#### 7.2.10. Genetical Approach

The primary measures to resolve any crop disease is the discovery of resistance in the respective disease, followed by the production of resistant varieties. This measure is also effective for KB disease management. After the 1940s, some cultivars of wheat have been documented as being resistant to KB. Based on the genetic basis of the interrogation, a large selection of wheat germplasms were evaluated at different centers in India and classified as resistant.

### 7.3. Models for KB Disease Predictions

“The Humid Thermal Index (HTI) model” is a tool for determining potential disease distribution. In the European Union for the KB Programme, this model was applied to assess the potential distribution of KB in Europe. Nagarajan [60] developed a “linear disease prediction model” to check the level of climatic suitability of KB infection by using average weekly weather. Diekmann [61] introduced “geophytopathology methodology” to create a link between the probability of KB with the difference between the average minimum and maximum temperature in the crop sowing month, the average minimum daily temperature in the winter months of the corresponding year, and the average maximum daily temperature at the wheat crop anthesis period. Subsequently, Diekmann [61] compared localities of disease prevalence and nonprevalence, which led to the development of the “GeoPhytopathology Model (GPMTI)” using Visual Basic and the Microsoft Access Database. It predicts the potential geographic area for KB pathogens using the collected climate data.

## 8. Revelation of Molecular Pathogenesis Mechanism by Multi-*Omics* Studies

Since the pathogen *Tilletia indica* Mitra is heterothallic fungus having a bipolar mating system, there is a scope of varied genetic recombination just before infection which mediates the pathogenesis mechanism. The advent of NGS and proteomics techniques have led to genomic dissection of the pathogen at various levels, paving way towards an understanding of the molecular facets of the pathogenicity [62]. The *Tilletia indica* genome is reported to have 97, 25, 63 and 7 effector linked, virulence triggering, loss of pathogenicity, and chemical resistance genes, respectively [63]. Besides this, [64] led to proteomics and genomics approaches to study the potential pathogenicity or virulence factors in this pathogen by developing the proteome map of its isolates at varied virulence. This study revealed the role of virulence proteins in host defense responses, cell wall degradation, piercing and adherence to host tissues, signal transduction pathway activation along with morphogenesis. Another secondary analysis using the existing genomic and transcriptomic data revealed additional seven genes contributing significantly to the pathogenesis mechanism in host penetration, causing infection [65].

For a large scale in-depth biological study, gene and genome sequences lead to meaningful information. The findings of several genomic studies have significantly deepened the knowledge of the genetic basis of KB disease among scientists. We reviewed the groundbreaking studies in KB pathogen genome sequencing in this section. Diverse genomic variations were detected with high precision in KB with the aid of next-generation sequencing technologies. The first draft genome sequence was published with two monosporidial lines of the KB pathogen Ti, covered the estimated size of 37.46 and 37.21 Mbp, from which 12,046 and 12,129 protein coding genes were predicted [66]. Subsequently, another genome sequence, known as TiK, was generated using the Illumina and PacBio sequencing platforms in 2017 [67] with draft assembly of 10,957 contigs and a total size of 26.7 Mb. The predicted number of putative genes was found 11,535 and was annotated to 31.77% of associated gene ontology terms, where about only 135 genes were identified with the pathogenic role. Various candidate pathogenicity/virulence factors has been reported from the secretome of this TiK isolate such as mannitol dehydrogenase (TiMD), secretory lipase (TiL), transaldolase (TiTA), enolase (TiE), phosphoglucoseisomerase (TiPGI), thioredoxin (TiTR), peptidyl prolyl isomerase (TiPPI), and aspartate protease (TiAP) [64]. Consequently, Mishra et al. [68] announced the first improved genome sequence of the monoteliosporic culture of Ti, consisting of the lowest number of 787 scaffolds with an estimated genome size of 31.83 Mbp, comprising of 9209 genes, of which 220 were virulence-associated genes (VAGs). Further, Gurjar et al. [63], assembled a genome size of 33.7 Mb with a 10,113 predicted genes. In the same year, Nguyen et al. [69], assembled the genome sequence of three isolates, one from India and another two from Pakistan in 29.7, 29.0 and 29.0 Mb, respectively, with 9410, 9677 and 9664 gene models, respectively. They also identified two species-specific single copy genes unique to Ti at the amino acid level. Currently, a total of nine genome sequence assemblies are reported but no database is available to date for the genome sequences of Ti studies (Table 2). There is large genome variation in the reported genome assemblies, which have a variable size range of 26.7 Mb to 43.7 Mb, which could be due to high number of segmental duplications, copy number variations and transposable elements. This fact indicates that the species Ti still remains challenging for genomic and genetic study. Further, no significant variation in mycelial growth and mycelial weight has been observed, but significant variation in sporulation has been observed between different Ti isolates [70].

The up-to-date genome assembly and annotation will assist in-depth study of host-defense and pathogen virulence, crop design with improved resistance and fungicide development by molecular modelling and simulation of numerous structures found in the genome and transcriptome based studies. The characterization of Ti isolates will help to understand the pathogenesis mechanism for developing novel Karnal bunt management strategies. With the large-scale publicly available genome and functional genomics, we expect to see impressive scientific findings in KB research, as well as fungicidal breakthroughs that will be useful for crop scientists and plant geneticists in future.

## 9. KB Resistant Quantitative Trait Loci (QTLs)/Genes Mapped in Host Wheat

The host plant resistance is the most effective and economic method for disease control. Indu-Sharma et al. [71] showed that resistance stability was expressed by 744 lines out of 43,680 bread wheat germplasm lines (*Triticum aestivum* L. emend. Fiore and Paol.) screened against Karnal Bunt disease, among which 188 strains had multiple resistance to Karnal Bunt, yellow (*P. striiformis*) rust and brown (Puccinia recondita Rox.ex. Desm.) rust. By pedigree breeding methods and pyramiding of KB resistant genes, three high yield KB resistant (W 7952, W 8618, W 8086) and six KB free wheats (KBRL 10, KBRL 15, KBRL 13, KBRL 22, KBRL 18, KBRL 24) have been developed, respectively. During the years 1995 to 2002, Kaur and Nanda [72] tested the wheat genotypes HD 2329, WH 542, WL 711, WL 1562, PBW 343, PBW 396 along with the resistant HD 29 genotype by artificial inoculum for sensitivity to KB disease. The findings revealed that the most vulnerable to KB was WL-711. Singh et al. [73], collected the resistant wheat varieties from The International Maize and Wheat Improvement Center, CIMMYT, Mexico and Advanced Varietal Trials were tested at potential geographic area for KB under artificially inoculated conditions during the years 1990/91 to 1993/94 in the Himachal Pradesh, Haryana, New Delhi, Punjab and Uttar Pradesh states of India. Eighteen genotypes (HP 1531, W 285, W 382, W 388, W 485, HD 29, HD 30, HD 2385, WL 1786, WL 6975, WL 7247, RAJ 2296, HW 502, ND 589, ND 602, PBW 34, PBW 225, DWL 5010) were the resistant lines out of the 66 inputs. KB was also resistant to 19 lines obtained from CIMMYT. The ICAR-National Bureau of Plant Genetic Resources (NBPGR), New Delhi had already registered both HD 29 and HD 30 as INGR 99012 and 99011, respectively. Brown-Guedira et al. [74], crossed HD 29 (resistant) and WL 711 (susceptible) wheat germplasm to develope 130 wheat recombinant inbred lines (RILs). When 81 AFLP and 90 SSRs loci were mapped in the RILs, approximately 1/3 of the disease response variation were collectively accounted by markers on Chr 2A, 4B and 7B. In three separate experiments, the genomic region on the long arm of Chr 4B showed the highest effect, where KB disease response reduced by half. GWM538, a closely linked SSR marker has been reported as a useful marker-assisted selection for KB resistance. A KB resistant wheat germplasm (KBRL 22) was developed from a cross of two resistant parent germplasms—“HD 29” and “W 485”—by Sharma et al. [75]. Following the artificial inoculations, the numbers of plants free from KB and affected by KB were collected from BC1, BC2, BC3, BC4 progenies and F2 generation. Singh et al. [76], examined KB resistant germplasm at Punjab Agricultural University, Ludhiana for three years and reported two new QTLs (Qkb.ksu-5 BL.1 and Qkb.ksu-6 BS.1) from HD 29 having resistant alleles, which were mapped at Xgdm116-Xwmc235 interval on Chr 5B and Xwmc105-Xgwm88 on Chr 6B. One other QTL (Qkb.ksu-4 BL.1) has been reported from ‘W 485’ having resistance allele mapped at Xgwm6-Xwmc349 interval on Chr 4B. The study of Hernandez et al. [77] for the identification of disease resistance QTLs, revealed the multitrait (MT) analysis as an effective approach rather than the individual QTL mapping analysis. They concluded RILs identified using MT analysis from “WH 542” and “HD 29” having combined resistance from multiple diseases. Brar et al. [78] identified four stable QTLs from their study from which QKb.cim-2BL, QKb.cim-3BS1 and QKb.cim-5BS2, QTLs were flanked by SNP marker. Two major clusters were identified on Chr 3B (Qkb.cnl-3B, QKb.cimmyt-3BS and Qkb.cim-3BS1) and Chr 4B (Qkb.ksu-4B, QKb.cimmyt-4BL, Qkb.cim-4BL) by Emebiri et al. [79]. Gupta et al. [80], reported one QTL on Chr 2BL in four experiments and another QTL on Chr 5BL in three experiments, which were having significant effects. They identified germplasm with <1% infection of KB, could be used for breeding to develop resistant cultivars.

## 10. Status of MAS/GWAS in Wheat for KB Genome

A genomewide association study on a collection of 179 prebreeding lines (PBLs) was conducted to determine genomic regions that confer resistance to KB disease in wheat [81]. This study revealed 15 significant variants (SNPs) associated to KB resistance from 6382 high quality DArTseq SNPs on Chr 2D, 3B, 4D and 7B, and were consistent with years, from which strong linkage disequilibrium (LD) was shown in two SNPs. Two candidate gene hits were identified as TraesCS4 D02 G350300 (a rhomboidlike protein belonging to the S54 family) and TraesCS4 D02 G352200 (TaNox8; a NADPH oxidase), while the computational analysis of SNPs on Chr 4D. A significant interaction between the loci of Chr 4D and 7B was found during the analysis of the epistatic interaction. They concluded that the resulted lines from Chr 4B could be used to integrate novel Karnal bunt resistance with any top cultivars worldwide. Further, 15 significant SNPs were reported on Chr 2D, 3B, 4D and 7B from combined analysis of the collected data of two years. This research is the first to identify KB resistance-associated QTLs on Chr 4D of wheat. Moreover, these findings gives new insight for further research aimed at improving Karnal bunt resistance.

## 11. Management Strategies for the Karnal Bunt

Due to the intermittent existence of the Ti pathogen of wheat, KB is difficult to control. Depending on the availability of favorable environmental conditions during headings, the occurrence of KB varies considerably from season to season. Since the spores can survive for long substantial periods of time in the soil, eliminating the fungus is very challenging. In wheat fields with high inoculum density, a substantial loss of quality and quantity typically occurs. Around 70 countries are therefore imposing quarantine restrictions on the wheat trade where KB is supposed to cause infection [82,83]. The effective management of KB, which is seed, soil and air borne, will rely on regulatory measures, host resistance, cultural practices, chemical measures, biosuppression techniques and their integration practices. The various measures to control KB are:

### 11.1. Use of Varieties Resistant to Karnal Bunt

The most effective approach to control this disease is the use of resistant varieties. To name a few, PBW 502 is the KB resistant wheat variety, while the N-75-3, N-75-5 and Pastour cultivars are partially KB resistant. The literature reports a number of wheat germplasms, their related genera and varieties resistant to KB disease [13,39,84].

### 11.2. Fungicide Seed Treatment

A number of chemical compounds have actively acted against Ti by reducing its teliospore germination. This approach is applicable to lower the transmission of inoculum by seeds, but only a few fungicides are currently approved for use against bunts. The active seed spores may be removed by this seed treatment, but if the seeds are planted in contaminated soil, they will not protect wheat plants from KB infection. It is necessary to use KB free seeds, but as the fungus is soil-borne, fungicide seed treatment is not entirely applicable for eliminating full infection, but it can reduce the probability of KB infection [85].

In order to remove the seed infection, seeds are soaked in Akh (*Calotropis procera*)/Eucalyptus (*E. globulus*)/Kali basuti (*Eupatorium adenophorum*)/Lantana (*L. camara*) around 250 mL/L for 1 h followed by drying. In general, it is used to combat other wheat bunt diseases, which is inadequate since it only controls the infection at the seedling level. For the removal of seed infections, other treatment includes Captan50 WP about 2.5 g/Kg, Dithane M-45 or, Vitavax 75 WP about 2.5 g/Kg, Kavach75 WP about 2 g/Kg Thiram 75 DS/Tilt 25 EC/Raxil 2 DS about 1 mL/Kg, Pseudomonas fluorescens of about 5 mL/Kg or *T. harzianum*/*Trichoderma viride* (Ecoderma) about 5 mL/Kg. The germination of teliospores has been prevented by cyano guanidine and hexachlorobenzene. Few agrochemical producers are looking for approval of fungicide against KB, e.g., carboxin with thiram (Vitavax 200/RTU-Vitavax-Thiram) and PCNB; however, it has been shown to limit the seed borne spores germination in Mexico [21]. The key problem of using these fungicides is that the spores of KB can germinate after the chemical has been washed off [86].

### 11.3. Fungicide Foliar Treatment

The regular application of fungicides is not a cost-effective practice. By applying foliar fungicides in between the late boot and flowering, the occurrence of KB can be considerably decreased. Agrozim, Bayleton, Carbendazim and Propioconazole are some of the useful fungicides. At the time of anthesis, propiconazole foliar fungicide spray should be sprayed as the disease is air-borne. Bavistin 50WP/Baycor 25WP/F-100/Moximate 72WP about 2.5 g/Kg, Contaf 25EC/Folicur 25ECor/Tilt 25EC about 1 mL/Kg foliar spray are applicable at the boot leaf stage. For the management of the KB of wheat [82], it is a cheaper and more environmentally friendly process.

### 11.4. Soil Fumigation

Soil fumigation with formaldehyde, methyl bromide and metam sodium like chemicals seems to have marginal effect in destroying teliospores. However, it is not cost-effective practice [87].

### 11.5. Biocontrol Agents

Approximately, full control over KB is provided by the integration of propiconazole with the *Trichoderma viride,* which is a bioagent fungus. It should be sprayed just before the emergence of the ear head, accompanied by chemical spray at the beginning of the emergence of the ear head [86].

### 11.6. Crop Rotation

To control KB disease, crop rotation can also be beneficial. It is also important to avoid dense crops. For up to five years, rotation in crops other than wheat crop is also required. This will increase the time lapse between wheat crops, further reducing the amount of teliospores in the soil. In order to control KB, rotation with nonhost crops can reduce spores [75]. In order to control the KB disease, crop rotation should be followed strictly as it has several advantages along with disease management [88,89].

### 11.7. Mulching

Polyethylene mulching can be used to increase temperature of soil and decrease the germination of teliospores. By use of polythene mulching activities and wheat straw burning after harvesting, it can raise the soil temperature by up to 54.5 °C. Polyethylene mulching and straw burning raise the soil temperature to 58.0 °C, 67.0 °C, 92.5 °C, at the soil depths of 10 cm, 5 cm and at the soil surface, respectively, which makes the soil disinfected from the teliospores [90].

### 11.8. Controlled Irrigation

In the heading and flowering period of wheat, proper irrigation and drainage is needed. Controlled irrigation, deep ploughing and planting of cover crops at the time of heading and flowering would help to mitigate KB disease [91]. Farmers need to consider a change in irrigation schedules to develop adequate conditions which could restrict the spread of pathogenic teliospores. It is also practicable to vary the dates of planting just to avoid the headings occurring in infectious weather conditions.

### 11.9. Changing Sowing Time

By avoiding the sowing of highly susceptible wheat cultivars and changing the sowing period in endemic areas, Karnal Bunt disease can be controlled. Changes in the sowing time can shift the flowering of the wheat from the infectious environmental conditions [90].

## 12. Major Researchable Gaps and Demand Driven Efforts in the Knowledge in KB Research

Taking into account the facts, the KB project’s primary objective is to provide unbiased, robust scientific data to support future risk analysis. Different research areas have concentrated on the assessment of the distribution and viability of teliospores. Strategic planning of the research requires improved methods for Ti detection and quantification to be developed through biotechnological interventions such as genetic variability and pathogenic studies, the integration of resistance in common wheat varieties, the development of germplasm markers for the identification of linked QTL associated with genes for the characteristics of disease resistance, and marker-assisted breeding with long-persistent resistance to KB. Whether resistance to Karnal bunt is controlled by a single major gene or expressed as a polygenic character has not been discovered so far due to the lack of a standardized population applied for screening the filial generations. In some of the commercial cultivars that are under large scale cultivation, resistance to KB needs to be improved with the characterization of the resistant genes and creating appropriate molecular markers for those genes. However, as Ti is heterothallic, an integrated analysis and retesting is necessarily required for the stability of the variants or races recognized by several research labs. In the future, it will also be appropriate to use computer-assisted software for risk analysis to monitor the KB disease. Moreover, many of the traits are governed by multiple QTLs/genes and transfers of such complex genes would be possible in future, where the degree of genetic comprehension is required, in multigene transfers. Unless strict quarantine is placed on the infected geographical areas, the pathogen is extremely difficult to eradicate. The infested area will need to be completely quarantined for many years with a ban on wheat cultivation. It would be important to regulate the movement of equipment and soil from the infested hot spot area. The farmers having affected fields should collaborate entirely to achieve success and for such co-operation they would need to be properly compensated for any loss by governments or regulatory agencies. In order to achieve enhanced detection of KB and wheat cultivars immune to KB, additional research is also required. One research area that should be targeted in KB research programs for effective disease forecasting and planning is the careful analysis of the pathogen and environmental relationship. Regulatory agencies should make efforts to meet the ultimate target of pathogen deregulation.

## 13. Conclusions

The present study is based on the analysis of published work from 1909 to 2020 (more than a centenary) in the field of KB disease. This overview highlights the systematic study of KB research and its potential for complementarity between risk assessments based on research. KB is a severe disease and has an effect on the global trade of wheat grains. Therefore, the efforts made in this study are the first attempt to quantify the overall scientific research on KB at one place. The intensive literature search in this study is unified at one place and will benefit the researchers with more reliable information. In deciding the most accurate course of action to avoid the spread of the fungi causing the disease, the proper identification, early detection and differential diagnosis of this fungal pathogen is of prime importance. Continuous efforts from scientists and farmers are needed to achieve successful control of this disease. Over the coming years, the development of more efficient and adaptable systems will continue to achieve higher sensitivity for high throughput KB detection. The management of KB disease could be accomplished by prohibiting contaminated seed material, encouraging farmers to grow bunt-resistant wheat varieties and creating awareness regarding the use of fungicides in KB-sensitive geographical areas. The best way to manage KB is genetic resistance, which is the most feasible method to control. While various resistant sources have been reported to date, very few genetic analyses have been conducted on them. An effective management of KB completely relies on our understanding explained in this paper and the integration of it into a suitable package of strategies. However, it is evident that efforts towards integrated disease management practices are feasible, leading to more secure and efficient disease management worldwide. Reading the past literature and having an understanding disease not only prompts us to remain alert but also gives us hope that we can find near-future solutions to control the Karnal bunt disease of wheat.

## Figures and Tables

**Table 1:** Morphologically similar teliospore details of *Tilletia* species.

*Tilletia* Species	Teliospore Size (µm)	Ornamentation Color	Surface View	Profile
*T. indica*	28–54	Densely tuberculate to narrowly cerebriform	Conical to truncate	Dark reddish brown to black, opaque
*T. inolens*	31–41	Coarse polygonal scales, made up of many fine spines	Coarse, cylindrical, flared and broken at tips	Dark golden yellow to dark chocolate brown
*T. cathcartae*	30–40	Densely verruculose to tuberculate	Cylindrical, truncate	Golden brown
*T. eragrostidis*	28–37	Coarse polygonal scales	Coarse truncate projections	Light to dark reddish brown
*T. walkeri*	23–45	Coarsely cerebriform to coralloid scales	Conical to truncate	Pale yellow to dark reddish-brown
*T. horrida*	17–36	Polygonal to cerebriform scales	Sharply pointed to truncate	Chestnut brown
*T. ehrhartae*	18–25	Coarse polygonal scales	Cylindrical, truncate	Olivaceous, dark
*T. rugispora*	17–28	Coarse polygonal scales	Conical, bluntly pointed	Mid-reddish brown
*T. barclayana*	14–36	Fine, polygonal to cerebriform scales	Sharply pointed to truncate	Chestnut brown

**Table 2:** List of genomes of *Tilletia* strains available till 2020.

S. No.	Assembly Name	GenBank Assembly ID	Submitter (Year)	Reference
1.	AAFC_TiDAOMC236408_1	GCA_009428345.1	Agriculture and Agri-Food, Canada (2019)	[69]
2.	AAFC_TiDAOMC236414_1	GCA_009428365.1		
3.	AAFC_TiDAOMC236416_2	GCA_001645015.2		
4.	ASM222083 v1	GCA_002220835.1	ICAR-IARI, India (2019)	[63]
5.	ASM299730 v1	GCA_002997305.1	GBPUAT, India (2017 and 2018)	[67]
6.	ASM305493 v1	GCA_003054935.1		[68]
7.	PSWKBGD_1_1	GCA_001689995.1	ICAR-IIWBR, India (2016)	[66]
8.	PSWKBGD_1_2	GCA_001689945.1		
9.	PSWKBGD_1_3	GCA_001689965.1		

## Data Availability

Not Applicable.
